# Supplementary material for: Enhancing Prostate Cancer Diagnosis with a Novel Artificial Intelligence-Based Web Application: Synergizing Deep Learning Models, Multimodal Data, and Insights from Usability Study with Pathologists
Source: Cancers (Basel). 2023 Nov 30;15(23):5659. doi: 10.3390/cancers15235659 (PMC10705310; doi:10.3390/cancers15235659)
Supplement: Supplementary file 1 [file cancers-15-05659-s001.zip › cancers-2650297-supplementary.pdf]

Questions for User Study
Prostate Cancer Web Application

Brief Introduction

The following set of questions aims to quantify usability, ease of understanding, accessibility, and acceptability of our web application to aid pathologists in grading prostate cancer H&E-stained Whole Slide Images (WSIs) of Biopsies.

The purpose of the application is to upload WSIs from a local device and obtain predicted ISUP grades powered by our state-of-the-art deep learning models along with additional metrics like demographic information, summarized information of the patient's latest clinical visit and clinical risk indicators.

Additionally, feedback questions are asked to help us improve aesthetic appeal and finetune the visualizations of our application to better suit the needs of pathologists.

- *The survey would take 10 – 15 minutes to complete, descriptive feedback is highly appreciated.*

Below is a screenshot of the main application page.

Figure S1: *Landing Page of the Web Application*

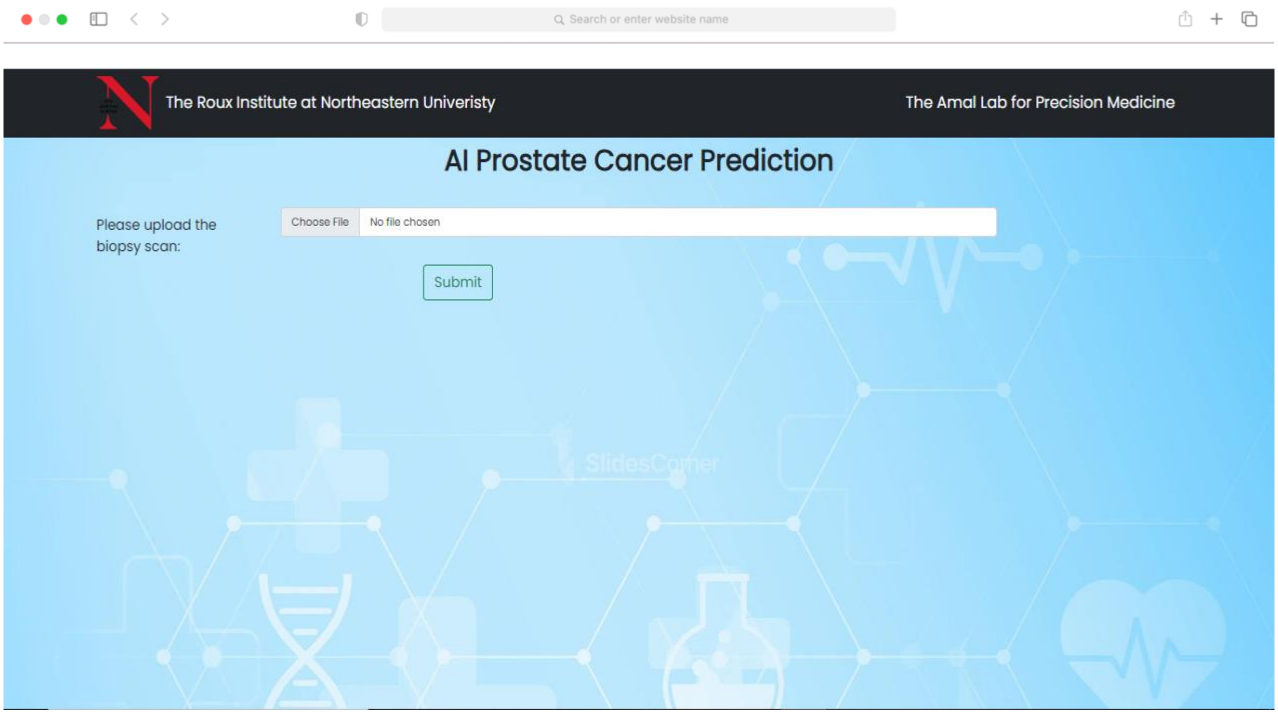

1. Do you have any suggestions to improve the landing page shown above in Figure S1?

Answer:

Questions Based on Page Displayed After Uploading the Biopsy WSI

Brief Overview: The donut chart shown on the left displays the predicted ISUP grade by averaging the results of all 5 backend deep learning models used. The right donut chart shows the probability distribution/ likelihood of each ISUP grade for 1/5 models based on the uploaded biopsy and displays the Grade with the highest probability in the title.

***Figure S2**: Output after Biopsy Image is Uploaded and Processed*

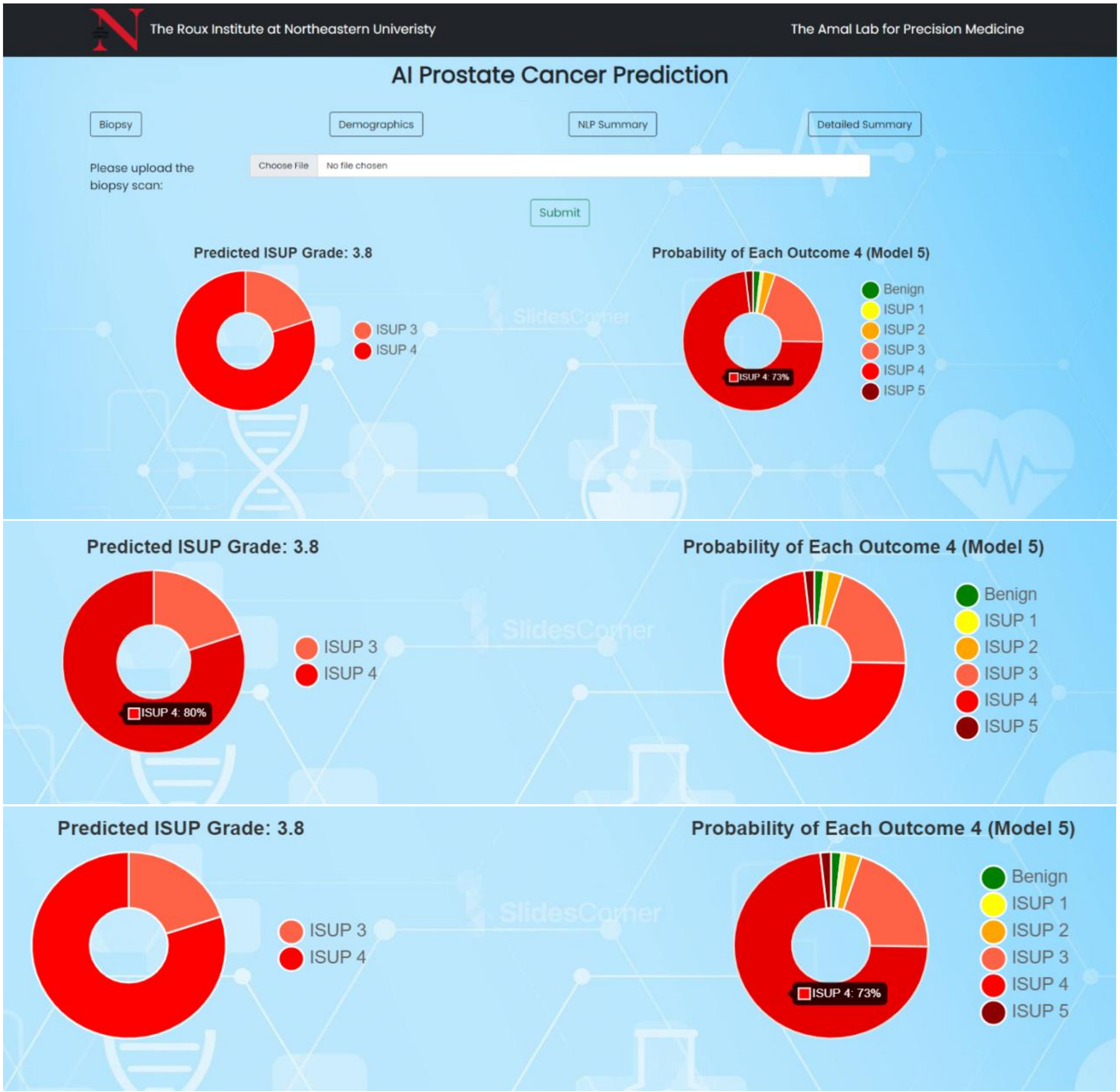

Answer the following questions on a scale of 1 to 7 based on Figure S2.

1. How well-defined is the primary goal of this visualization?

Answer:

2. To what extent does this visualization effectively convey its message?

Answer:

3. How easy is it to interpret the data in this visualization?

Answer:

4. How clearly could you differentiate between the two donut charts shown in Figure S2?

Answer:

5. Is the model probability output for all ISUP grades (right side) confusing to understand?

Answer:

6. How visually appealing is this visualization?

Answer:

7. How effectively does this visualization communicate information through color, shape, and other visual elements?

Answer:

8. How understandable are the labels and axes used in this visualization?

Answer:

9. How useful is this visualization and related tabs (demographics, biopsy, etc) for its intended purpose?

Answer:

10. How frustrated/stressed did you feel while understanding the visualizations and interacting with the web application?

Answer:

Based on Figure S2 Answer the Following Descriptive Questions

Do you have any ideas on how to increase the value or potency of this visualization?

Answer:

Tab Specific Questions

1. Biopsy Tab

Figure S3: Biopsy Pop-Up When Biopsy Button is Clicked

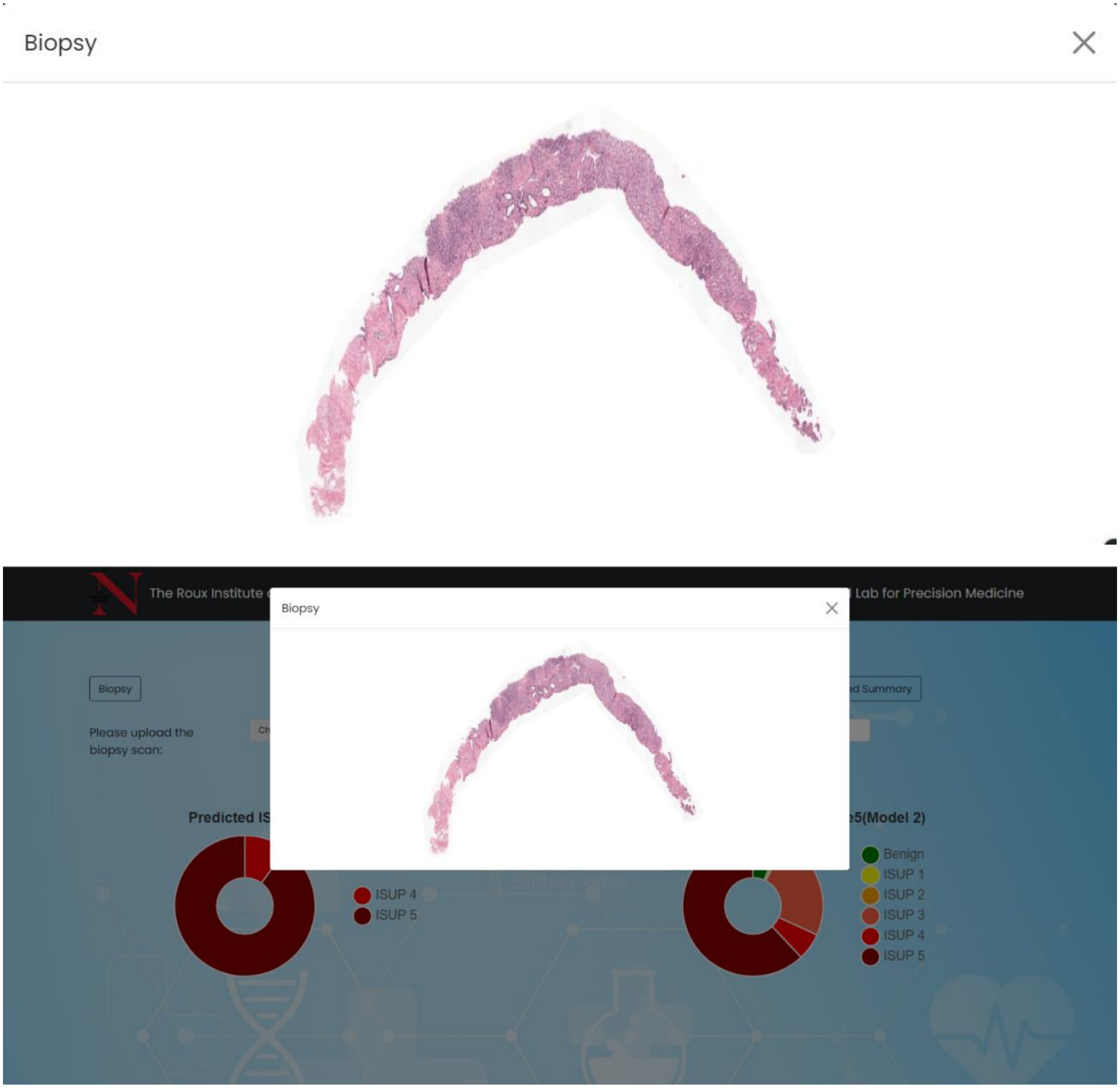

Answer the following questions related to Figure S3 on a scale of 1 to 7

1. How helpful is displaying the biopsy along with predicted results for cancer grading process?

Answer:

2. How good is the quality of the biopsy image shown in the figure?

Answer:

3. Does seeing the biopsy with the predicted results add any bias to your decision-making process?

Answer:

Answer the following questions in Yes/No format related to Figure S3

1. Would you consider grading the biopsy from the web app itself if the highest image resolution is provided?

Answer:

2. Does seeing the biopsy with a predicted grade potentially reduce grading time?

Answer:

Any suggestions to improve the visualization of the biopsy?

Answer:

2. Demographics Tab

Figure S4: Basic Demographic Information of The Patient

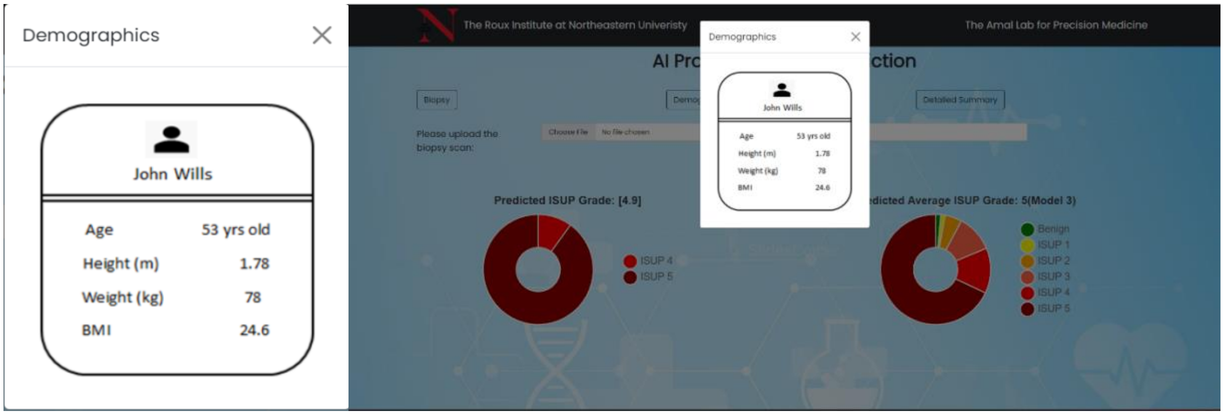

Answer the following questions on a scale of 1 – 7 related to Figure S4.

1. To what extent does the webpage effectively provide demographic information?

Answer:

2. How well does the webpage present risk factors, including risks for different diseases?

Answer:

3. How helpful Is this information for Prostate Cancer Grading?

Answer:

Descriptive Questions (Related to Figure S4)

1. Is there any additional information you would like to see along with what has already been shown?

Answer:

2. Do you have any suggestions for improving this visualization?

Answer:

3. NLP (Natural Language Processing) Summary Tab

Figure S5: NLP Summarization of Patient Information (If Available)

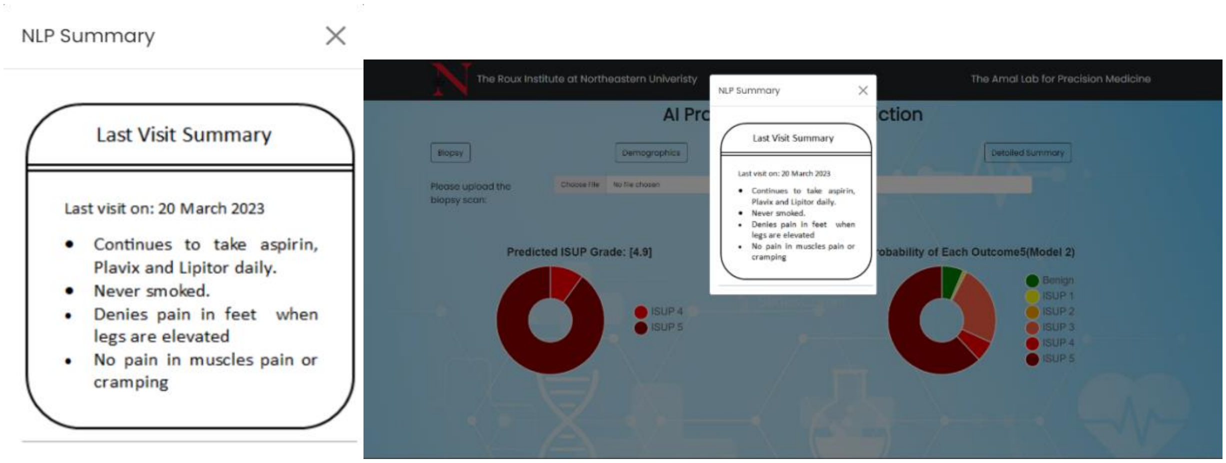

Answer the following questions on a scale of 1 – 7 related to Figure S5.

1. How Effectively Does the NLP summary button provide a concise and informative summary of the patient's condition?

Answer:

2. How useful is this information for prostate cancer diagnosis?

Answer:

4. Detailed Summary Tab

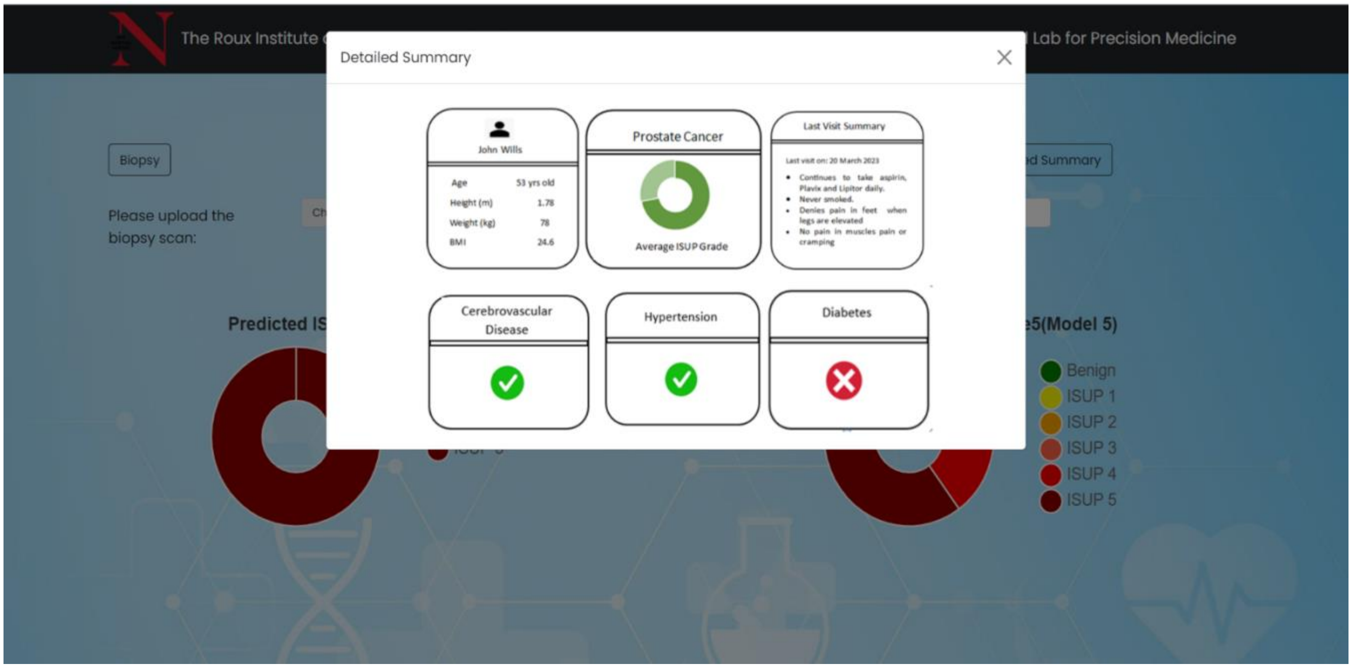

1. Do you prefer seeing all the information shown together?

Answer:

Overall, Web Application – Feedback Questions

Answer the following questions on a scale of 1 to 7

1. How comprehensive and detailed is the patient summary provided on the webpage?

Answer:

2. How helpful is the webpage in providing relevant information to the user?

Answer:

3. Does the webpage adequately address the user's needs for understanding demographics, risk factors, and patient summaries?

4. How well does the webpage cater to diverse levels of user expertise or knowledge?

Answer

5. How visually appealing is the design and layout of the webpage?

Answer:

6. Are the buttons and elements on the webpage intuitive and easy to navigate?

Answer:

7. Does the overall visual presentation enhance the user's experience and engagement with the webpage?

Answer:

Finally, answer the next 6 questions on a scale of 1 (Low) to 20 (High) based on NASA (National Aeronautics and Space Administration) Task Load Index format to help us assess the overall usability, acceptance, and workload experienced throughout this process.

Note: Lower scores will be considered better for all 6 categories

1. Mental demand- how much thinking, deciding, or calculating was required to perform the task.

Answer:

2. Physical demand - the amount and intensity of physical activity required to complete the task.

Answer:

3. Temporal demand - the amount of time pressure involved in completing the task.

Answer:

4. Effort - how hard does the participant have to work to maintain their level of performance?

Answer:

5. Performance - the level of success in completing the task/one full interaction with the web app.

Answer:

6. Frustration level - how insecure, discouraged, or secure or content the participant felt during the task.

Answer:
